# Phenolic Compounds of Olive Oil: Intake and Behavior in In Vitro Static Simulated Digestion Without or with Cellulose

**DOI:** 10.3390/molecules31162784

**Published:** 2026-08-10

**Authors:** Lidija Jakobek, Petra Matić, Lidija Šoher, Daniela Kenjerić

**Affiliations:** Faculty of Food Technology Osijek, Josip Juraj Strossmayer University of Osijek, Franje Kuhača 18, HR 31000 Osijek, Croatia; petra.matic@ptfos.hr (P.M.); lidija.soher@ptfos.hr (L.Š.); daniela.kenjeric@ptfos.hr (D.K.)

**Keywords:** bioaccessibility, recovery, gastrointestinal tract, cellulose, food matrix

## Abstract

The effects of phenolic compounds in the gastrointestinal tract are linked to their intake in the diet and their interactions with the food matrix. The aim was to estimate the intake of phenolic compounds from two olive oils and to assess their behavior in the gastrointestinal tract after simulated static in vitro digestion without or with added cellulose, as a preliminary result on the influence of cellulose on their behavior. Individual phenolic compounds were quantified by RP-HPLC. The two olive oils were characterized by phenyl alcohols (1.8% and 5.3%), flavones (1.1% and 2.4%), lignans (0.8% and 1.7%), and unidentified secoiridoid derivatives (96.3% and 90.7%). The estimated population daily intake of phenolic compounds based on EFSA consumption data for the analyzed olive oil samples had means 0.75 to 1.27 and 0.30 to 0.51 mg per day, with the 95 percentile up to 5.91 and 2.38 mg per day for olive oil 1 and 2, respectively. The total amounts after gastric and intestinal digestion significantly decreased. Phenolic subgroups showed different behavior. In the stomach, phenyl alcohols increased, while all other subgroups decreased. After the intestinal phase, some subgroups additionally increased (phenyl alcohols, flavones, lignans), while others decreased (secoiridoids). Cellulose increased recovery in the stomach. As a preliminary result, the influence of cellulose needs to be studied further.

## 1. Introduction

Olive oil is a major component of the Mediterranean diet [1], with many beneficial effects demonstrated in previous studies. In a study evaluating olive oil consumption and cause-specific mortality in the Spanish population, olive oil was associated with a decreased risk of overall mortality and a significant reduction in cardiovascular disease mortality [2]. Olive oil has also shown antidiabetic activity [3,4], a protective effect against hyperlipidemia [5], and many positive effects in the gastrointestinal tract [6,7,8]. Olive oil provided gastric protection [7], enhanced the antioxidant profile of the small intestine [6], and exerted an anti-inflammatory effect on inflammatory bowel disease [8]. These and similar findings have brought considerable attention to olive oil and the elucidation of its potential benefits.

Some protective effects of olive oil in the gastrointestinal tract are attributed to phenolic compounds [4,8], their antioxidant and anti-inflammatory activities, and metabolic actions [4]. Olive oil contains various phenolic compounds, including phenyl alcohols, flavones, secoiridoid derivatives, and lignans [9,10,11,12,13,14]. To understand beneficial effects in the gastrointestinal tract, it is important to consider the behavior of olive oil phenolic compounds during digestion and their interactions with other constituents of the food matrix. The behavior during digestion has been studied [15,16,17,18,19,20,21]. Some studies have also examined interactions between olive oil phenolics and food matrices. Vitali Čepo et al. (2020) [20] investigated how various food matrices, or components such as cellulose, pectin, and inulin, interact with olive oil and affect the behavior and accessibility for absorption of hydroxytyrosol, tyrosol, and total phenolics from olive oil in the gastrointestinal tract. Interactions between olive oil and meat during digestion have also been studied [19]. However, knowledge about how interactions between constituents of the food matrix and phenolic compounds from olive oil affect the release and final recovery of phenolics in different parts of the gastrointestinal tract remains insufficient. Recovery refers to the amount of phenolic compounds released from the food matrix during digestion compared with their total amount in food. Interactions with food constituents can influence the recovery of phenolic compounds and consequently their beneficial activity in the gastrointestinal tract.

Cellulose is a component of many plant-based foods, such as lettuce and various vegetables, which are often consumed with olive oil in a Mediterranean-style diet. However, to the best of our knowledge, there are not enough studies on the interactions between cellulose and phenolic compounds from olive oil. The interaction of some phenolic compounds, such as catechin, caffeic acid, and ferulic acid, with cellulose has been studied previously [22]. Moreover, earlier works investigated the formation of complexes of phenolic compounds from olive oil with cellulose derivatives such as ethyl cellulose [23], cellulose-based hydrogels [24], magnesium-grafted bacterial cellulose [25], different dietary fibers like pectin [26], or strawberry dietary fibers or cellulose [27]. The aim of such studies was to formulate carriers of phenolic compounds through the gastrointestinal tract [24,27], after which they can be released in the colon and exert beneficial effects. Another aim was to use such complexes in the formulation of functional foods [23,26], or in dermatological and medicinal applications [24,25]. However, to the best of our knowledge, the interaction of major phenolic compounds from olive oil with cellulose during digestion, and how those interactions affect the recovery and behavior of phenolic compounds, has not been elucidated.

The first aim of this study was to characterize the main phenolic compounds in two olive oils and their pomace left after oil production, and to estimate the intake of phenolic compounds and oils. The second aim was to conduct in vitro static simulated gastrointestinal digestion of olive oils without cellulose and with two different levels of cellulose, to assess the effect of cellulose on the behavior and recovery of phenolic compounds during digestion.

## 2. Results and Discussion

### 2.1. Phenolic Compounds in Olive Oils and Pomace and the Intake

Phenolic compounds were identified based on their UV/Vis spectra and retention times (Table S1, Figure S1). Representative chromatograms of olive oil and pomace with identified compounds are shown in Figure S2. Five individual phenolic compounds were identified in two olive oils (Table 1): phenyl alcohols (hydroxytyrosol, tyrosol), lignans ((+)-pinoresinol), and flavones (luteolin, apigenin). For the secoiridoid derivatives that were not identified, the amount was estimated for the entire group together. A total of seven compounds were identified in the pomace remaining after olive oil production (Table 2):

The amounts of phenolic compounds in olive oils 1 and 2 are shown in Table 1. The olive oils contained 5.7 and 6.6 mg kg^−1^ of hydroxytyrosol, 3.7 and 4.3 mg kg^−1^ of tyrosol, 3.9 and 3.4 mg kg^−1^ of (+)-pinoresinol, 4.3 and 4.2 mg kg^−1^ of luteolin, and 1.3 and 0.7 mg kg^−1^ of apigenin. Similar amounts of these compounds were found in earlier studies [12,13,16] and in [9,10,14]. In particular, low- and high-price extra virgin olive oils and olive oils contained hydroxytyrosol up to 18.7 mg kg^−1^, tyrosol up to 24.5 mg kg^−1^, (+)-pinoresinol up to 10.3 mg kg^−1^, luteolin up to 4 mg kg^−1^, and apigenin up to 1.9 mg kg^−1^ [14]. Those results are consistent with the results of this study. Another study also found similar amounts of these compounds [9]. In that study, hydroxytyrosol was found in extra virgin olive oils up to 4.1 mg kg^−1^ and tyrosol up to 1.57 mg kg^−1^. (+)-Pinoresinol was present in oils up to 4.1 mg kg^−1^, apigenin up to 0.82 mg kg^−1^, and luteolin up to 2.3 mg kg^−1^ [9]. The concentrations of hydroxytyrosol, tyrosol, and pinoresinol in 55 different olive oil samples ranged from 0.3 to 29.3, 3.6 to 38.4, and 1.4 to 13.9 mg kg^−1^, respectively [10], consistent with the results here. Moreover, the oils contained various unidentified secoiridoid derivatives, with amounts of 492 and 186 mg kg^−1^. In the study by Ricciutelli et al. (2017) [14], the amount of unidentified secoiridoid derivatives together with hydroxytyrosol and tyrosol ranged from 126 to 414 mg kg^−1^ in high- and low-price extra virgin olive oils, which agrees with the results of this study.

Pomace remaining after olive oil production is still rich in various phenolic compounds (Table 2). The total amount of phenolic compounds in olive oil pomace ranges from 176 to 754 mg kg^−1^. Hydroxytyrosol, tyrosol, oleuropein, and ligstroside were found in amounts ranging from 12.3 to 126.3, 19.4 to 84.8, 50.2 to 171.9, and 50.8 to 123.6 mg kg^−1^, respectively. (+)-Pinoresinol, luteolin, and apigenin were present in amounts of 9.2 to 19.8, 29.3 to 214.7, and 4.9 to 13.9 mg kg^−1^, respectively. These amounts are consistent with an earlier study [28].

**Figure 1:**
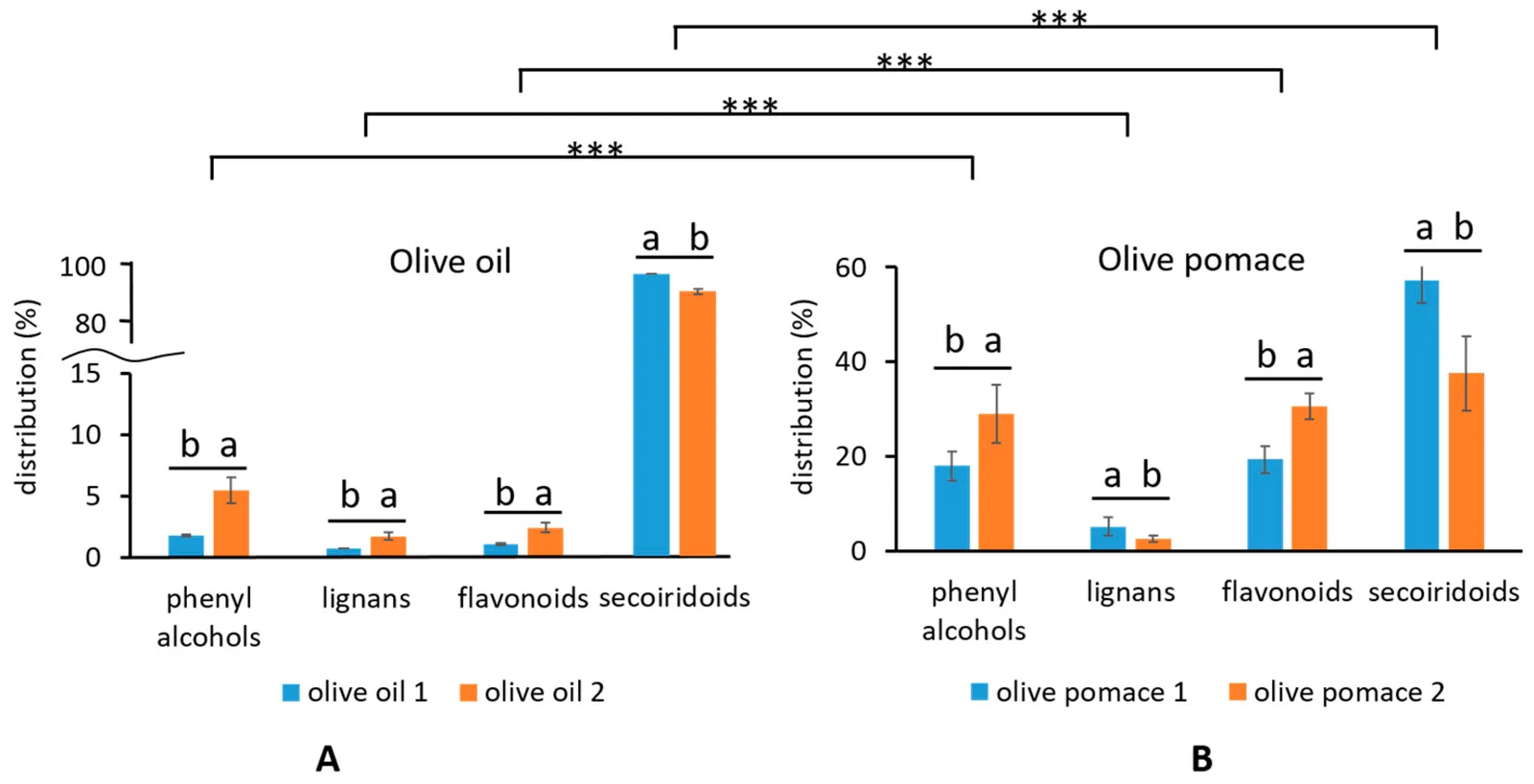
The percentage distribution of phenolic compounds in (**A**) olive oils and (**B**) pomace. Olive oil and pomace samples were analyzed in triplicate, each measured 2 times with HPLC (*n* = 6). Different letters indicate a significant difference in phenolic subgroups between olive oil 1 and 2, or pomace 1 and 2, determined by a post hoc Tukey test at 95% significance. *** indicates a significant difference between phenolic subgroups in olive oils and pomace determined by a *t*-test at 99.9% significance.

Figure 1 shows the distribution of phenolic subgroups in olive oils and pomace. Secoiridoid derivatives represented the highest proportion of total phenolic compounds in oils 1 and 2, at 96.3% and 90.7%, respectively, while other phenolic groups contributed only small percentages (lignans 0.8% and 1.7%, phenyl alcohols 1.8% and 5.3%, flavones 1.1% and 2.4%). The difference in the distribution of phenolic subgroups between the two olive oils was significant (*p* < 0.05).

In pomace, the distribution was more even (Figure 1): phenyl alcohols, lignans, and flavones accounted for 18.0% and 28.0%, 5.2% and 2.6%, and 19.4% and 30.3%, respectively. Secoiridoids accounted for 57.4% and 39.1%. Again, the difference in the distribution of phenolic subgroups in the two pomaces was significant (*p* < 0.05). Another visible difference is between samples: olive oil is statistically different from olive pomace (*p* < 0.001).

To further characterize olive oils, olive oil intake was estimated using the EFSA database (European Food Safety Authority) (Table 3). Depending on the population group, mean olive oil intake ranged from 1.47 to 2.49 g per day, with the upper endpoint of the 95% interval at 11.58 g per day. Based on these data, and the measured amounts of phenolic compounds, the estimated daily intake of phenolic compounds ranged from 0.75 to 1.27 mg per day for olive oil 1, and from 0.30 to 0.51 mg per day for olive oil 2, depending on the group, with the upper endpoint of the 95% interval reaching 5.91 and 2.38 mg per day, respectively.

### 2.2. Phenolic Compounds After the Simulated Digestion of Olive Oils

Chromatograms of samples after digestion with identified compounds are shown in Figure S3.

Gastric digestion: Table 1 presents the amounts of individual phenolic compounds identified after gastric digestion. The same individual compounds were identified after gastric digestion as in the original olive oils, except for apigenin, which was not found. The flavone apigenin diminished during gastric digestion, as reported by Suárez et al. (2010) [29], consistent with our results. The amounts of the individual phenyl alcohols hydroxytyrosol and tyrosol increased after gastric digestion of both olive oils compared to the amounts before digestion. Similar results were previously reported [18], with an increase in hydroxytyrosol, a decrease in tyrosol, and an increase in hydroxytyrosol acetate. Suárez et al. (2010) [29] also reported increases in both compounds after gastric digestion. The amounts of unidentified secoiridoid derivatives, the lignan (+)-pinoresinol, and the flavone luteolin decreased.

**Figure 2:**
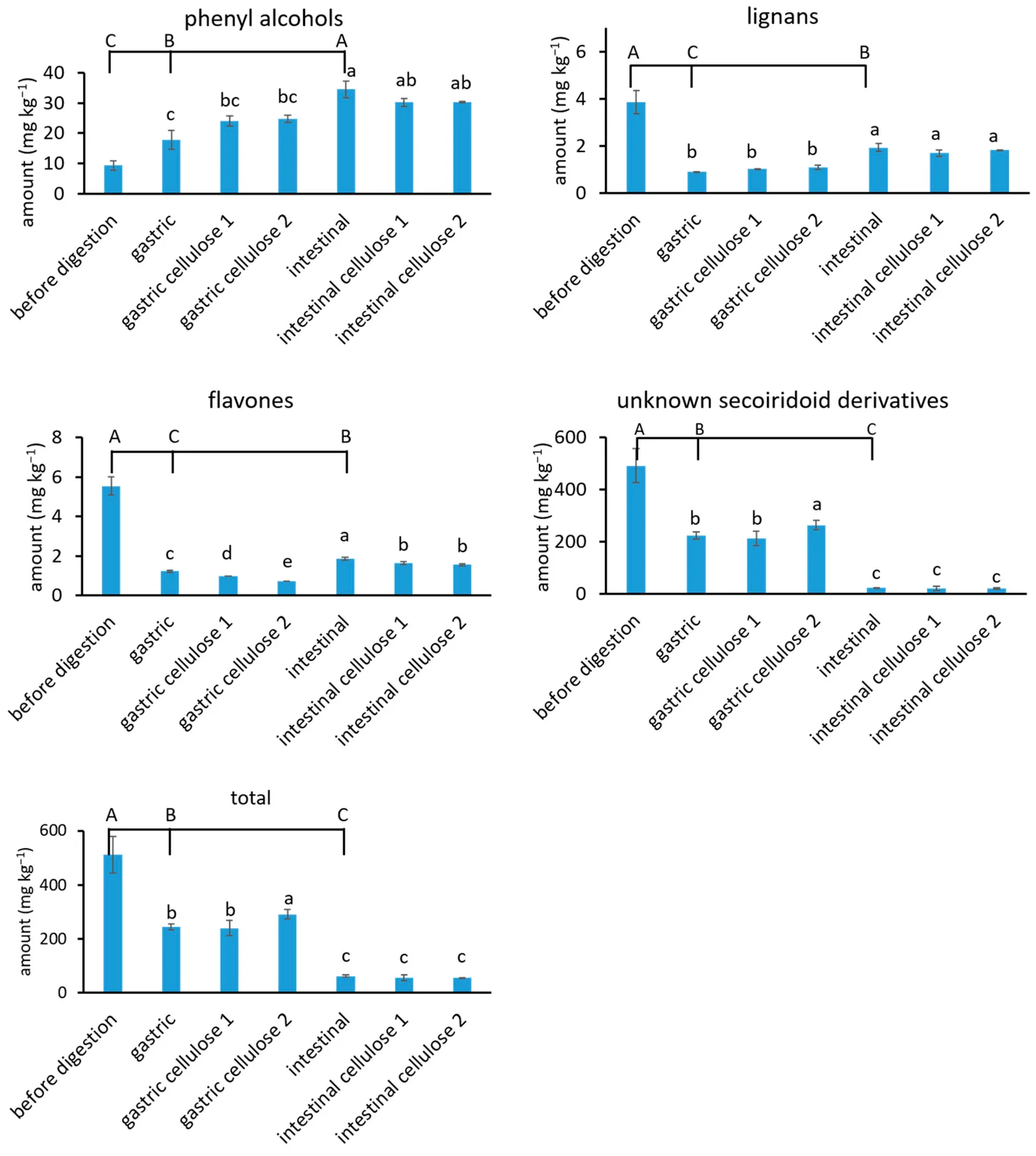
Phenolic compound subgroups before and after gastric and intestinal digestion of olive oil 1. Digestion was conducted without or with the addition of cellulose at two levels (cellulose 1 and cellulose 2). A first post hoc Tukey test analyzed differences in phenol amounts before digestion and after gastric and intestinal digestion, with differences indicated by different uppercase letters. A second post hoc Tukey test analyzed differences in phenol amounts after gastric and intestinal digestion with or without added cellulose, with differences indicated by different lowercase letters.

**Figure 3:**
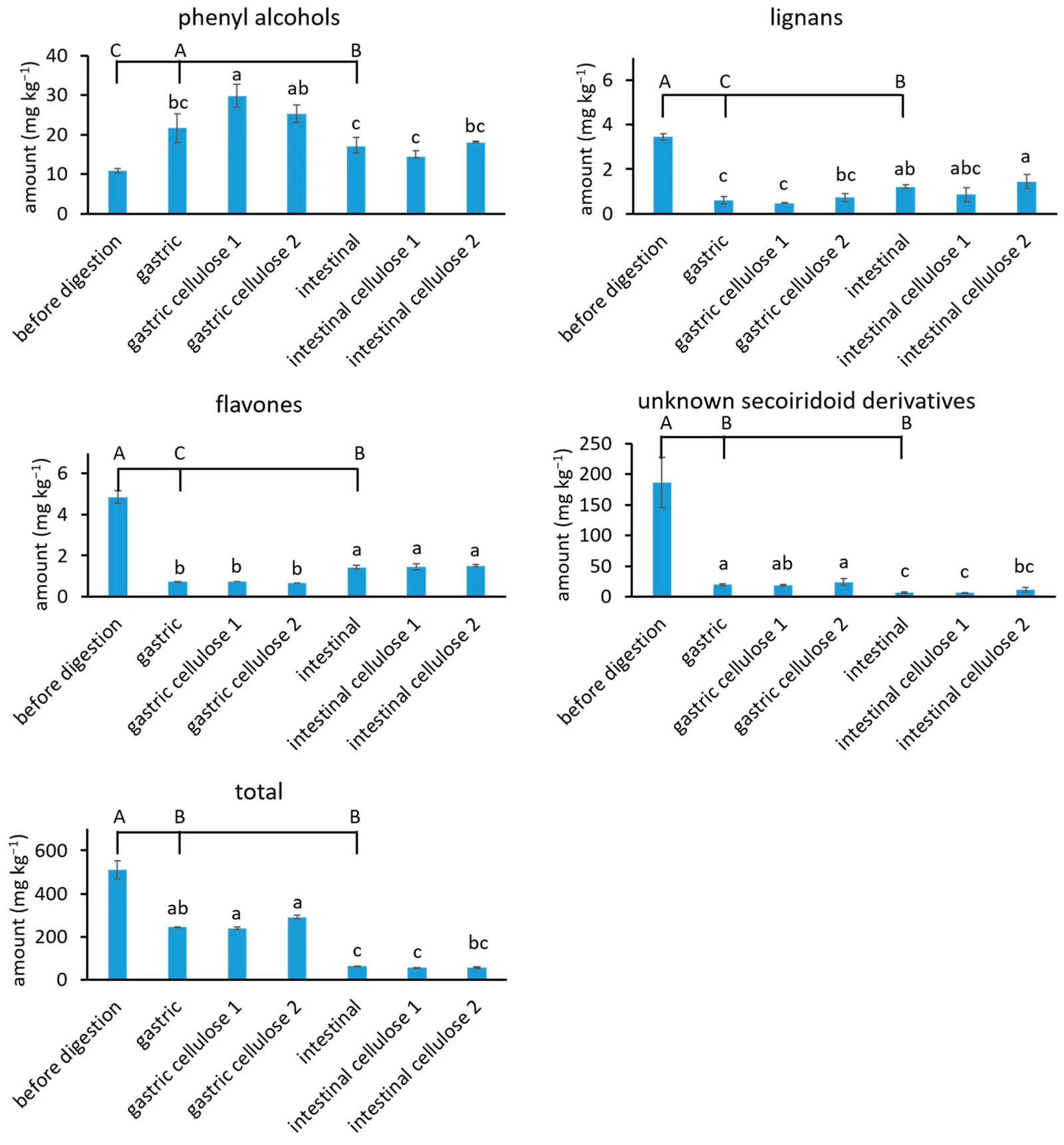
Phenolic compound subgroups before and after gastric and intestinal digestion of olive oil 2. Digestion was conducted without or with the addition of cellulose at two levels (cellulose 1 and cellulose 2). A first post hoc Tukey test analyzed differences in phenol amounts before digestion and after gastric and intestinal digestion, with differences indicated by different uppercase letters. A second post hoc Tukey test analyzed differences in phenol amounts after gastric and intestinal digestion with or without added cellulose, with differences indicated by different lowercase letters.

Figure 2 and Figure 3 show the total amounts of specific subgroups before digestion and after gastric digestion of olive oils 1 and 2, respectively. The subgroups follow the same trend as the individual compounds. The total amounts of phenyl alcohols increased after gastric digestion of both olive oils compared to the amounts before digestion. The amounts of unidentified secoiridoid derivatives, lignans, and flavones in both oils decreased. The increase in phenyl alcohols [18,29] and the decrease in secoiridoids [18,29,30], have been reported previously. The decrease in lignans and flavones is also consistent with the findings of Reboredo-Rodríguez et al. (2021) [18] and Suárez et al. (2010) [29]. Overall, the total amounts of phenolic compounds decreased after gastric digestion compared to the oils before digestion.

The increase in simple phenyl alcohols shown here could be due to the intense hydrolysis of secoiridoid derivatives under gastric digestion conditions. The amount of secoiridoids did decrease after gastric digestion. Hydrolysis produced higher amounts of hydroxytyrosol and tyrosol. Earlier studies have shown that secoiridoid derivatives hydrolyze at pH 2 of the gastric phase, releasing hydroxytyrosol [31].

Secoiridoids (91.8% and 45.9% in oils 1 and 2, respectively) and phenyl alcohols (7.3% and 51.5% in oils 1 and 2, respectively) accounted for a high percentage of the total recovered amount. The results of this study are based on in vitro digestion and only two oil samples, so they need to be confirmed using a dynamic digestion model and in vivo studies.

Intestinal phase: Table 1 shows the amounts of individual phenolic compounds after intestinal digestion. The amounts of hydroxytyrosol and tyrosol in the first oil significantly increased compared to the gastric phase. In the second oil, hydroxytyrosol was not detected, while the amount of tyrosol significantly increased compared to the gastric phase. In an earlier study, the amount of tyrosol increased and hydroxytyrosol decreased [29], similar to this study. At the same time, secoiridoid derivatives in both oils significantly decreased or were completely absent compared to the gastric phase. The lignan (+)-pinoresinol and the flavone luteolin in both oils significantly increased. Luteolin can be stable under small intestinal conditions, and both luteolin and pinoresinol increased in the small intestinal phase according to earlier studies [18,29], similar to this study.

Figure 2 and Figure 3 show the total amounts of specific subgroups recovered after intestinal digestion of olive oils 1 and 2, respectively. In the first oil, the amount of total phenyl alcohols significantly increased after the small intestinal phase compared to the gastric phase, whereas in the second oil, total phenyl alcohols decreased. An increase in phenyl alcohols was also reported by Reboredo-Rodríguez et al. (2021) [18] and Vitali Čepo et al. (2020) [20]. Similarly, in another study, the total amount of phenyl alcohols increased in the small intestine [29]. All these earlier results are generally consistent with our findings. Secoiridoid derivatives in both oils significantly decreased or were completely absent compared to the gastric phase. Lignans and flavones in both oils significantly increased. Overall, the total amount of phenolic compounds significantly decreased from the gastric phase to the end of intestinal digestion.

The results suggest that secoiridoid derivatives were not stable under small intestinal conditions, leading to their hydrolysis and a lower amount. As a result, the amount of phenyl alcohols formed during hydrolysis may have increased. Moreover, in the first olive oil, oleuropein was found after the intestinal phase, which may also result from the hydrolysis of secoiridoids.

Secoiridoids accounted for a high percentage (37.5% and 25.8% in olive oils 1 and 2, respectively) and, together with phenyl alcohols (56.2% and 64.4% in olive oils 1 and 2, respectively), they constituted a large portion of the total phenolics in the small intestine. However, these results are based only on an in vitro static digestion model, and on two olive oils, and as already mentioned, dynamic digestion models and in vivo studies are necessary to confirm these findings.

### 2.3. The Cellulose and Phenolic Compounds in Digestion

Some differences were observed in the released compounds after gastric digestion with or without cellulose (Figure 2 and Figure 3). Cellulose was added at two levels: level 1 (0.005 g) and level 2 (0.01 g) per 0.25 mL of olive oil. With the addition of cellulose to olive oil 1, the amounts of phenyl alcohols (hydroxytyrosol and tyrosol), unknown secoiridoids, and total phenolics increased compared to gastric digestion without cellulose, in some cases significantly. Only the amount of flavones (luteolin) significantly decreased. Lignans mainly remained unchanged compared to digestion without cellulose. In olive oil 2, the same increase in phenyl alcohols, and total phenolics with the addition of cellulose was observed, but the differences were not significant.

In the small intestine, a slight decrease was observed when cellulose was added to oil 1 (Figure 2 and Figure 3), with the decrease in flavones (luteolin) being significant. No visible differences were observed after small intestinal digestion of olive oil 2. Earlier results reported a similar decrease with the addition of cellulose [20].

It can be suggested that adsorption processes [22,23] might have occurred in the simulated digestion of olive oils with cellulose. In our earlier studies, we investigated the adsorption of pure phenolic compounds [32], phenolic compounds from apples [33,34], or aronia [35] onto β-glucan, a soluble dietary fiber. Pure phenolic compounds and apple or aronia phenolic compounds were indeed adsorbed onto β-glucan in model solutions at pH 5.5 [32,33], pH 1.5, 3, and 7 [35], or in simulated gastric and intestinal digestion fluids [34]. Other causes of changes in phenolic compounds during digestion, according to earlier studies, might include interactions with digestive enzymes and bile salts [26]. As the influence of cellulose described here represents preliminary results, further studies are necessary to confirm these suggestions.

### 2.4. Apparent Recovery of Phenolic Compounds

Apparent recovery was calculated as the percentage released from the food matrix during digestion, compared to the amount present before digestion. Table 4 presents recovery values for phenolic subgroups. In the stomach, phenyl alcohols from both oils showed the highest potential, apparent recovery (190% and 199%) due to secoiridoids whose breakdown formed additional amounts of phenyl alcohols. Phenyl alcohols were followed by secoiridoid derivatives (46% and 11%), lignans (23% and 18%), and flavones (22% and 15%). Total recovery ranged from 21% to 48%.

In the small intestine, the apparent recovery of phenyl alcohols from both oils was 367% and 156%. The high percentages of recovered phenyl alcohols are probably the result of the previously mentioned breakdown of secoiridoids. The apparent recovery values are consistent with a previous report, where they ranged from 172% to 4186% for individual phenolic alcohols [18]. Another earlier study reported recovery of hydroxytyrosol and tyrosol of 86% and 99%, respectively [17], which is lower than in our study. Furthermore, the apparent recovery of lignans was 50% and 35%, and that of flavones was 34% and 30%. The values are similar to those reported earlier for individual lignans (25% to 59%) and flavones (23% to 54%) [18] or reported values were lower (luteolin 7%, apigenin not identified) [17]. The apparent recovery of secoiridoids was low (5% and 4%). Secoiridoid compounds undergo significant losses in the gastric and duodenal regions due to degradation and formation of tyrosol and hydroxytyrosol [36], which agrees with this study. Total apparent recovery reached 13%, which was significantly lower than the amount recovered in the stomach.

## 3. Materials and Methods

### 3.1. Chemicals and Samples

Standards of tyrosol (79058, ≥99.5%) and oleuropein (92167, ≥98%) were purchased from Sigma-Aldrich (St. Louis, MO, USA), and luteolin (89245) from Phytolab (Vestenbergsgreuth, Bavaria, Germany). Hydroxytyrosol (4999S, 98%), apigenin (1102S, 99%), ligstroside (0240, ≥90%), and (+)-pinoresinol (8642, ≥95%) were purchased from Extrasynthese (Genay, France). Methanol (HPLC grade) was obtained from J.T. Baker (Gliwice, Poland), acetonitrile (HPLC grade) from Fisher Scientific (Loughborough, UK) and hexane from Carlo Erba reagents (Val de Reuil Cedex, France). Chemicals for simulated digestion, such as α-amylase 13 U mg^−1^ (A3176), pepsin 695 U mg^−1^ (P7000), pancreatin 8 USP (P7545), and bile salt (B8756), were purchased from Sigma-Aldrich (St. Louis, MO, USA). Potassium chloride, potassium dihydrogen phosphate, sodium hydrogen carbonate, calcium chloride, and magnesium chloride were purchased from Gram mol d.o.o. (Zagreb, Croatia), ammonium carbonate from Kemika (Zagreb, Croatia), and sodium chloride from Carlo Erba Reagents (Val de Reuil, France).

Stock solutions of standard phenolic compounds were prepared in methanol and diluted with methanol to obtain lower concentrations for calibration curves. Stock solutions of electrolytes for simulated digestion were prepared in 0.5 mol L^−1^ (KCl, KH_2_PO_4_, (NH_4_)_2_CO_3_), 1 mol L^−1^ (NaHCO_3_), 2 mol L^−1^ (NaCl), and 0.15 mol L^−1^ (MgCl_2_). Simulated digestion fluids were prepared from these electrolytes by pipetting aliquots into a final volume of 100 mL. The final concentrations in mmol L^−1^ in simulated salivary fluid (SSF) were 18.9 KCl, 4.6 KH_2_PO_4_, 17 NaHCO_3_, 0.056 MgCl_2_, and 0.06 (NH_4_)_2_CO_3_; in simulated gastric fluid (SGF), 8.6 KCl, 1.1 KH_2_PO_4_, 31.3 NaHCO_3_, 0.15 MgCl_2_, 0.63 (NH_4_)_2_CO_3_, and 59 NaCl; and in simulated intestinal fluid (SIF), 8.5 KCl, 1 KH_2_PO_4_, 106.3 NaHCO_3_, 0.4 MgCl_2_, and 48 NaCl. The pH of SSF, SGF, and SIF was adjusted to 7, 3, and 7, respectively, with 1 mol L^−1^ HCl. Enzymes were prepared in SSF (α-amylase 1500 U mL^−1^), SGF (pepsin 20,000 U mL^−1^), and SIF (pancreatin 800 U mL^−1^). Bile salts were prepared in SIF (25,000 mg L^−1^).

Olives were harvested in 2025 from a local orchard in Croatia. Olive oil was prepared in mills using traditional milling and cold pressing techniques. Olive oil 1 was produced from Pedolino and Leccino cultivars, while olive oil 2 was made from Pedolino, Leccino, and Levantinka cultivars. The obtained oil was packed in dark green bottles and sealed. Olive pomace was placed in plastic bags and transported to the laboratory. It was then dried in a commercial dryer (KYS-328A, Delimano, Guandong Kangye Electric Appliances, Foshan City, China), and packed in vacuum-sealed bags.

### 3.2. Extraction of Phenolic Compounds

Olive oil (0.25 mL) and hexane (0.25 mL) were pipetted into a plastic tube. Then, 1.2 mL of 80% methanol in water was added to the tube. The tube was vortexed, placed in an ultrasonic bath for 30 min (RK-100, Bandelin electronic GmbH, Berlin, Germany), and then centrifuged at 10,000 rpm for 10 min (Eppendorf Minispin, Eppendorf, Hamburg, Germany). The alcoholic layer was pipetted into a new plastic tube. The residue was extracted again using the same procedure, and the alcoholic extracts were combined, evaporated under vacuum in a vacuum concentrator (Concentrator plus, Eppendorf, Hamburg, Germany) (30 min, 45 °C), and centrifuged (10,000 rpm, 10 min). Methanol (200 μL) was added to the extract, and the extract was filtered through a PTFE syringe filter, before HPLC analysis.

Phenolic compounds from oil pomace (0.12 g) were extracted with the same procedure.

### 3.3. Simulated Digestion of Olive Oil

The digestion was conducted according to Minekus et al. (2014) [37] with some modifications. To obtain the sample after gastric digestion, olive oil (0.25 mL) was pipetted into a plastic tube. SSF (175 μL), H_2_O (48.8 μL), CaCl_2_ (1.25 μL), and α-amylase (25 μL) were added to the tube. The tube was vortexed and placed in a dry block thermostat (Bio TDB-100, Biosan, Riga, Latvia) for 2 min at 37 °C (salivary digestion). Next, SGF (375 μL), H_2_O (14.8 μL), CaCl_2_ (0.25 μL), HCl 1 M (10 μL), and pepsin (100 μL) were pipetted into the tube. The tube was vortexed and placed in the dry block thermostat for 2 h at 37 °C (gastric digestion). The tube was then placed in an ice water bath for 5 min, centrifuged for 10 min at 10,000 rpm, and the aqueous layer was pipetted into a new plastic tube. Hexane (300 μL) was added, the sample was centrifuged for 10 min at 10,000 rpm, and the aqueous layer was filtered with a PTFE syringe filter before HPLC analysis.

To obtain the sample after small intestinal digestion, the procedure was the same as for gastric digestion, with the small intestinal digestion following. After salivary and gastric digestion, SIF (550 μL), H_2_O (180.5 μL), CaCl_2_ (0.3 M, 2 μL), NaOH 1 M (7.5 μL), pancreatin (250 μL), and bile salt (10 μL) were added to the tube. The tube was vortexed and placed in the dry block thermostat for 2 h at 37 °C (intestinal digestion). The tube was then placed in an ice water bath for 5 min, centrifuged for 10 min at 10,000 rpm, and the aqueous layer was pipetted into a new plastic tube. Hexane (300 μL) was added, the sample was centrifuged for 10 min at 10,000 rpm, and the aqueous layer was filtered with a PTFE syringe filter before HPLC analysis.

Gastric and intestinal digestion of olive oil was also conducted with the addition of cellulose using the same procedure. Cellulose was added at two levels: level 1 (0.005 g) and level 2 (0.01 g).

Recovery represents the percentage of phenolic compounds measured at the end of the digestion phase, relative to the amount extracted from oil before digestion (%). It was calculated according to the following equation:

(1) $$(\mathrm{recovery})(\%)=\frac{\gamma_{\mathrm{digestion}\;\mathrm{phase}}(\mathrm{mg}{\mathrm{kg}}^{-1})}{\gamma_{\mathrm{before}\;\mathrm{digestion}}(\mathrm{mg}{\mathrm{kg}}^{-1})}100$$

where γ_digestion phase_ is the amount of phenolic compounds at the end of the digestion phase (mg kg^−1^ oil), and γ_before digestion_ is the amount of phenolic compounds before digestion (mg kg^−1^ oil).

### 3.4. HPLC Analysis

Samples after extraction and simulated digestion were analyzed on an Infinity II HPLC system equipped with a quaternary pump, PDA detector, vial sampler, Poroshell 120 EC-C18 column (4.6 × 100 mm, 2.7 μm), and Poroshell 120 EC-C18 4.6 mm guard column (Agilent Technologies, Santa Clara, CA, USA). Mobile phases A and B were 0.1% H_3_PO_4_ in water and acetonitrile, respectively. The mobile phase flow rate was set to 0.4 mL min^−1^, with a gradient program as follows: 0 min 5% B, 0 to 5 min 5 to 30% B, 5 to 15 min 30 to 40% B, 15 to 20 min 40 to 43% B, 20 to 30 min 43 to 53% B, 30 to 32 min 53 to 90% B, 32 to 34 min 90 to 5% B, 34 to 36 min 5% B. Linearity of calibration curves was expressed as r^2^ (from 0.9980 to 0.9996). LOD and LOQ were as follows: hydroxytyrosol 0.39 and 1.21 mg kg^−1^, tyrosol 0.01 and 0.02 mg kg^−1^, (+)-pinoresinol 0.21 and 0.50 mg kg^−1^, luteolin 0.18 and 0.54 mg kg^−1^, apigenin 0.06 and 0.17 mg kg^−1^, oleuropein 0.50 and 1.53 mg kg^−1^, ligstroside 0.53 and 1.60 mg kg^−1^. Spectra were collected from 190 to 600 nm, and chromatograms were scanned at 280 and 320 nm. Phenolic compounds were identified by spiking olive oil and pomace extracts with known standards, and by comparing UV/Vis spectra and retention times with those of standards. Calibration curves of standards were used for quantification. Unknown secoiridoid derivatives were detected with the help of literature [9,14,38] and UV/Vis spectra, and quantified using the calibration curve of oleuropein. Oleuropein was chosen since many derivatives identified in earlier studies [9,14,38] are oleuropein based.

### 3.5. Estimated Daily Intake

Phenolic compound intake was estimated at the age-group level using average daily olive oil consumption data, including 95th percentile intake, obtained from the EFSA database [39] and measured concentrations of total phenolic compounds in two analyzed olive oil samples (205.4 and 510.9 mg kg^−1^), providing a potential range of polyphenol intake.

### 3.6. Statistical Analysis

This study included two biological replicates (two olive oils and two pomaces). Each of the two olive oils and two olive pomaces was extracted three times. Each extract was analyzed twice by HPLC to determine the amounts of phenolic compounds in olive oils 1 and 2 and pomaces 1 and 2 before digestion (*n* = 6). Gastric and small intestinal digestion of each olive oil was performed in two parallel experiments, each measured twice by HPLC (*n* = 4). Digestion with cellulose was performed once, with each measured twice by HPLC (*n* = 2). Results were expressed as mean ± standard deviation. The amounts were analyzed with the post hoc Tukey test using Statistica 13.3 (Cloud Software Group, Inc., Fort Lauderdale, FL, USA). Microsoft Excel (Redmond, WA, USA) was used for the *t*-tests.

## 4. Conclusions

The intake of phenolic compounds, estimated with the EFSA database, is not high and is based on only two olive oils, but it represents a normal diet. Phenolic compounds were released from the oil matrix in the gastric and small intestinal phases of digestion. After gastric digestion, the total amount of phenolic compounds decreased compared to the amounts in the oils, and from the gastric phase to the end of the intestinal phase, the total amounts decreased further. Secoiridoids and phenyl alcohols accounted for a high percentage of the compounds in both the gastric and intestinal phases. The preliminary results on the influence of cellulose were not very pronounced. Since this study is limited to a static in vitro digestion model, studies under dynamic digestive conditions and in in vivo models are needed. Future studies should also include more olive oil samples and a precise characterization of all phenolic compounds in olive oil and during digestion, using different and more accurate analytical methods. Further studies that could also be suggested are investigations of the influence of different amounts of cellulose, different food sources of cellulose or different food matrices on the release and recovery of phenolic compounds from olive oil.

## Figures and Tables

**Table 1:** The amount (mg kg^−1^ of oil) of phenolic compounds in olive oil, and after the gastrointestinal digestion of olive oils.

	Phenyl Alcohols		Secoiridoids	Lignan	Flavones		Unknown	
	Hydroxytyrosol	Tyrosol	Oleuropein	(+)-Pinoresinol	Luteolin	Apigenin	Secoiridoid Derivatives	Total
Olive oil 1								
Before digestion	5.7 ± 1.0	3.7 ± 0.6	n.d.	3.9 ± 0.5	4.3 ± 0.5	1.3 ± 0.1	492.2 ± 66.0	511.1 ± 68.8
Gastric	10.4 ± 1.1 ^b^	7.4 ± 2.1 ^b^	n.d.	0.9 ± 0.0 ^b^	1.2 ± 0.1 ^c^	n.d.	224.1 ± 13.1 ^a^	244.0 ± 10.2 ^b^
Gastric cellulose 1	14.9 ± 1.2 ^a^	9.1 ± 0.6 ^b^	n.d.	1.0 ± 0.0 ^b^	1.0 ± 0.0 ^d^	n.d.	213.3 ± 27.0 ^a^	239.3 ± 28.8 ^b^
Gastric cellulose 2	16.1 ± 0.4 ^a^	8.7 ± 1.6 ^b^	n.d.	1.1 ± 0.1 ^b^	0.7 ± 0.0 ^e^	n.d.	264.2 ± 18.9 ^a^	290.8 ± 17.6 ^a^
Intestinal	14.3 ± 1.6 ^a^	20.1 ± 2.2 ^a^	23.0 ± 1.7 ^a^	1.9 ± 0.2 ^a^	1.9 ± 0.1 ^a^	n.d.	n.d.	61.2 ± 4.5 ^c^
Intestinal cellulose 1	9.6 ± 0.4 ^b^	20.6 ± 0.9 ^a^	21.2 ± 9.5 ^a^	1.7 ± 0.1 ^a^	1.6 ± 0.1 ^b^	n.d.	n.d.	54.7 ± 10.8 ^c^
Intestinal cellulose 2	12.7 ± 0.3 ^ab^	17.5 ± 0.6 ^a^	21.1 ± 1.8 ^a^	1.8 ± 0.0 ^a^	1.6 ± 0.1 ^b^	n.d.	n.d.	54.7 ± 1.5 ^c^
Olive oil 2								
Before digestion	6.6 ± 0.4	4.3 ± 0.2	n.d.	3.4 ± 0.2	4.2 ± 0.3	0.7 ± 0.0	186.2 ± 41.0	205.4 ± 42.1
Gastric	11.8 ± 1.4 ^b^	9.9 ± 2.3 ^b^	n.d.	0.6 ± 0.2 ^c^	0.7 ± 0.0 ^b^	n.d.	19.5 ± 2.0 ^a^	42.5 ± 2.6 ^ab^
Gastric cellulose 1	17.8 ± 0.8 ^a^	12.1 ± 2.1 ^ab^	n.d.	0.5 ± 0.0 ^c^	0.8 ± 0.0 ^b^	n.d.	18.8 ± 1.7 ^ab^	50.0 ± 4.6 ^a^
Gastric cellulose 2	16.2 ± 1.0 ^a^	9.0 ± 1.3 ^b^	n.d.	0.7 ± 0.2 ^bc^	0.7 ± 0.0 ^b^	n.d.	24.1 ± 5.4 ^a^	50.7 ± 7.9 ^a^
Intestinal	n.d.	17.0 ± 1.6 ^a^	n.d.	1.2 ± 0.1 ^ab^	1.4 ± 0.1 ^a^	n.d.	6.8 ± 1.1 ^c^	26.4 ± 1.4 ^c^
Intestinal cellulose 1	n.d.	14.4 ± 0.4 ^ab^	n.d.	0.9 ± 0.3 ^abc^	1.5 ± 0.1 ^a^	n.d.	6.5 ± 0.6 ^c^	23.3 ± 0.8 ^c^
Intestinal cellulose 2	n.d.	17.9 ± 0.0 ^a^	n.d.	1.4 ± 0.3 ^a^	1.5 ± 0.0 ^a^	n.d.	11.5 ± 3.5 ^bc^	32.3 ± 3.7 ^bc^

Olive oils were analyzed in triplicate, each measured 2 times with HPLC (*n* = 6). Digestion was conducted in duplicate, each measured 2 times with HPLC (*n* = 4). Digestion with cellulose was conducted once, each measured 2 times with HPLC (*n* = 2). Different letters in a column indicate a significant difference determined by a post hoc Tukey test at 95% significance. n.d. not detected.

**Table 2:** The amount (mg kg^−1^) of phenolic compounds in olive pomace.

	Phenyl Alcohols		Secoiridoids		Lignan	Flavones		Total
	Hydroxytyrosol	Tyrosol	Oleuropein	Ligstroside	(+)-Pinoresinol	Luteolin	Apigenin	
Pomace 1	12.3 ± 1.2	19.4 ± 3.9	50.2 ± 13.9	50.8 ± 6.0	9.2 ± 3.4	29.3 ± 3.9	4.9 ± 1.4	176.1 ± 33.5
Pomace 2	126.3 ± 33.7	84.8 ± 22.8	171.9 ± 107.4	123.6 ± 73.0	19.8 ± 7.9	214.7 ± 50.9	13.9 ± 3.8	755.5 ± 299.4

Olive pomace was analyzed in triplicate, each measured 2 times with HPLC (*n* = 6). phenyl alcohols (hydroxytyrosol, tyrosol), secoiridoids (oleuropein, ligstroside), lignans ((+)-pinoresinol), and flavones (luteolin, apigenin).

**Table 3:** Estimated olive oil intake (mean values and 95th percentile, in g per day) and estimated daily intake of phenolic compounds (mean phenolic compound intake and 95th percentile, in mg per day) from olive oil according to population groups based on EFSA consumption data and analyzed olive oil samples.

Population Group							
	Infants (3 m–1 y)	Toddlers (1–2 y)	Children (3–9 y)	Adolescents (10–17 y)	Adults (18–64 y)	Elderly (65–74 y)	Very Elderly (≥75 y)
Number of subjects	322	535	963	258	896	511	275
Consumers (%)	59.01	60.75	49.53	37.21	44.76	41.68	33.09
	Mean olive oil intake						
Olive oil intake	1.51 ± 2.30	1.85 ± 2.87	1.47 ± 2.52	1.50 ± 3.29	2.49 ± 4.42	2.34 ± 4.39	1.49 ± 3.45
95th percentile	6.03	7.40	6.95	7.66	11.43	11.58	7.02
	Estimated mean phenolic compound intake						
Olive oil 1	0.77 ± 1.17	0.94 ± 1.47	0.75 ± 1.29	0.77 ± 1.68	1.27 ± 2.26	1.20 ± 2.24	0.76 ± 1.76
95th percentile	3.08	3.78	3.55	3.91	5.84	5.91	3.58
Olive oil 2	0.31 ± 0.47	0.38 ± 0.59	0.30 ± 0.52	0.31 ± 0.67	0.51 ± 0.91	0.48 ± 0.90	0.31 ± 0.71
95th percentile	1.21	1.52	1.43	1.57	2.35	2.38	1.44

m = months, y = year; EFSA European Food Safety Authority.

**Table 4:** Apparent recovery of phenolics in the gastric and intestinal digestion (%).

Digestion	Phenyl Alcohols %	Lignans %	Flavones %	Secoiridoids %	Total %
	Olive oil 1				
gastric	190.0 ± 33.1 ^c^	22.9 ± 0.4 ^b^	22.2 ± 1.0 ^c^	45.5 ± 2.7 ^b^	47.8 ± 2.0 ^b^
gastric cellulose 1	256.3 ± 18.7 ^bc^	26.3 ± 0.5 ^b^	17.7 ± 0.2 ^d^	43.3 ± 5.5 ^b^	46.8 ± 5.6 ^b^
gastric cellulose 2	264.1 ± 12.9 ^bc^	28.1 ± 2.1 ^b^	13.0 ± 0.0 ^e^	53.7 ± 3.8 ^a^	56.9 ± 3.5 ^a^
intestinal Intestinal cellulose 1	367.4 ± 29.8 ^a^	49.9 ± 4.1 ^a^	33.5 ± 1.5 ^a^	4.7 ± 0.3 ^c^	12.0 ± 0.9 ^c^
intestinal cellulose 1	321.6 ± 13.5 ^ab^	43.8 ± 3.7 ^a^	29.3 ± 1.3 ^b^	4.3 ± 1.9 ^c^	10.7 ± 2.1 ^c^
intestinal cellulose 2	322.1 ± 3.1 ^ab^	46.8 ± 0.6 ^a^	28.0 ± 1.1 ^b^	4.3 ± 0.4 ^c^	10.7 ± 0.3 ^c^
	Olive oil 2				
gastric	198.6 ± 33.6 ^bc^	17.9 ± 4.9 ^c^	15.1 ± 0.3 ^b^	10.5 ± 1.1 ^a^	20.7 ± 1.3 ^ab^
gastric cellulose 1	273.8 ± 26.5 ^a^	13.9 ± 0.6 ^c^	15.5 ± 0.1 ^b^	10.1 ± 0.9 ^ab^	24.3 ± 2.2 ^a^
gastric cellulose 2	231.8 ± 21.1 ^ab^	20.9 ± 5.1 ^bc^	13.8 ± 0.1 ^b^	12.9 ± 2.9 ^a^	24.7 ± 3.8 ^a^
intestinal Intestinal cellulose 1	156.3 ± 14.5 ^c^	35.3 ± 2.7 ^ab^	29.6 ± 1.8 ^a^	3.6 ± 0.6 ^c^	12.9 ± 0.7 ^c^
intestinal cellulose 1	132.2 ± 3.9 ^c^	25.1 ± 9.1 ^abc^	30.2 ± 3.0 ^a^	3.5 ± 0.3 ^c^	11.3 ± 0.4 ^c^
intestinal cellulose 2	164.5 ± 0.2 ^bc^	41.9 ± 9.1 ^a^	31.1 ± 1.0 ^a^	6.2 ± 1.9 ^bc^	15.8 ± 1.8 ^bc^

Digestion without cellulose was conducted in duplicate, each measured 2 times with HPLC (*n* = 4). Digestion with cellulose was conducted once, each measured 2 times with HPLC (*n* = 2). Different letters in a column indicate a significant difference determined by the post hoc Tukey test (95% significance). The post hoc test was performed separately for olive oil 1 and olive oil 2.

## Data Availability

The raw data supporting the conclusions of this article will be made available by the authors on request.

## Supplementary Materials

The following supporting information can be downloaded at: https://www.mdpi.com/article/10.3390/molecules31162784/s1, Figure S1: UV/Vis spectra of standard phenolic compounds; Figure S2. Chromatograms of (A) olive oil 1 and 2, and (B) pomaces 1 and 2 left after the production of those oils, scanned at 280 nm, with identified compounds; Figure S3. An example of a chromatograms of olive oil after (A) gastric digestion, and (B) intestinal digestion, scanned at 280 nm, with identified compounds; Table S1: UV/Vis spectra of phenolic compounds identified in olive oil and pomace.
