# Supplementary material for: Phenolic Compounds of Olive Oil: Intake and Behavior in In Vitro Static Simulated Digestion Without or with Cellulose
Source: Molecules. 2026 Aug 10;31(16):2784. doi: 10.3390/molecules31162784 (PMC13515034; doi:10.3390/molecules31162784)
Supplement: Supplementary file 1 [file molecules-31-02784-s001.zip › molecules-4345303-supplementary.pdf]

Supplementary Materials

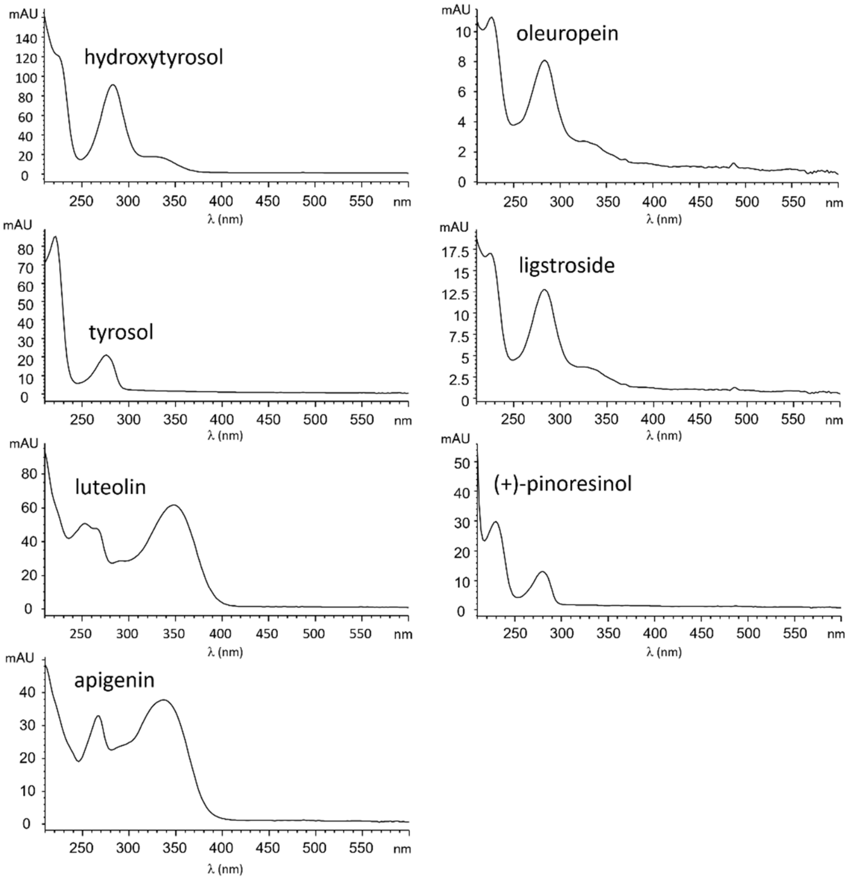

Figure S1. UV/Vis spectra of standard phenolic compounds.

Table S1. UV/Vis spectra of phenolic compounds identified in olive oil and pomace.

Compound	λ<br>nm
hydroxytyrosol	280
tyrosol	222, 276
luteolin	254, 268, 348
apigenin	266, 338
oleuropein	284
ligstroside	284
(+)-pinoresinol	230, 280

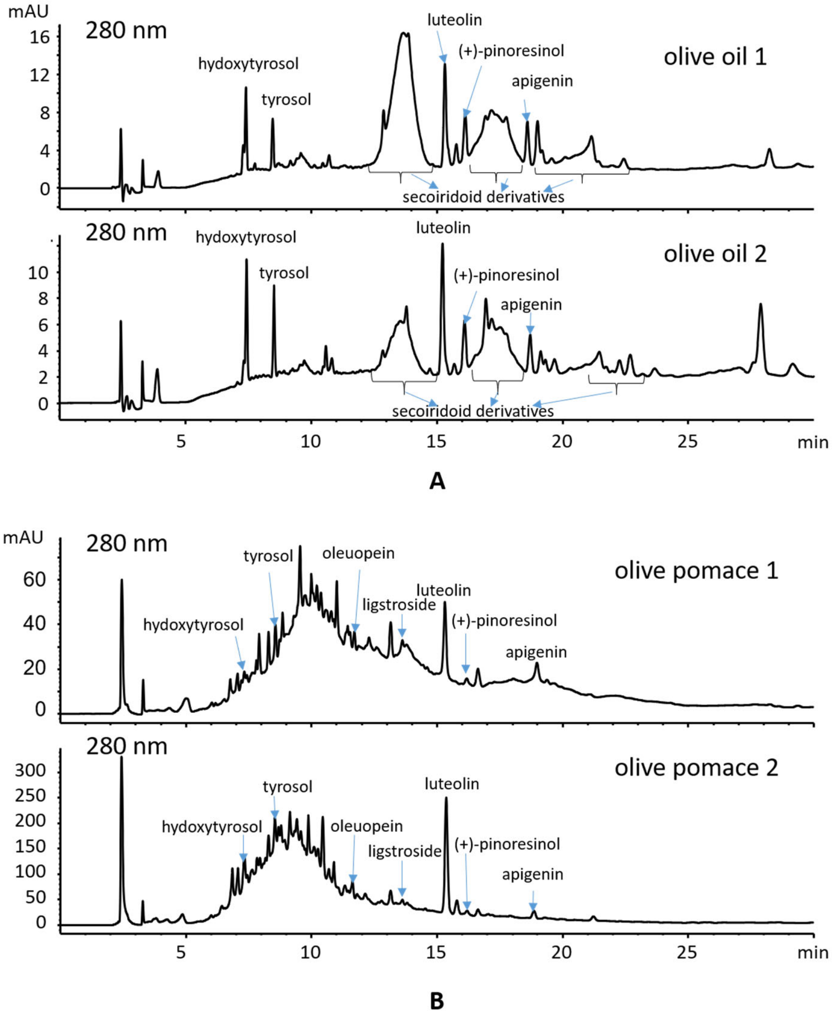

Figure S2. Chromatograms of A) olive oil 1 and 2, and B) pomaces 1 and 2 left after the production of those oils, scanned at 280 nm, with identified compounds.

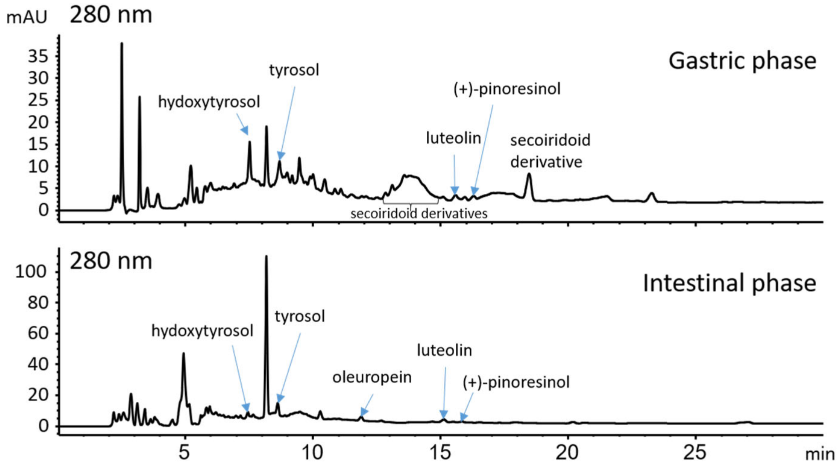

Figure S3. An example of a chromatograms of olive oil after A) gastric digestion, and B) intestinal digestion, scanned at 280 nm, with identified compounds.
